# Management of intra-bony defects using nano HA+PRF over its combination with a diode laser

**DOI:** 10.6026/973206300211108

**Published:** 2025-05-31

**Authors:** Saurabh Gupta, Pravin M. Parmar, Ravinder Singh, Nitika Gupta, Lubna Tabassum Siddiqui, Shahbaz Asif Ali, Shail D. Kushwaha, Abhigyan Manas

**Affiliations:** 1 Private Practitioner, Periodontist and Implantologist, Jammu, Jammu and Kashmir, India; 2 Prosthodontist, Gujarat University, Dental Surgeon Class 1, General Hospital Botad, Gujarat, India; 3 Department of Oral Medicine and Radiology, Maharishi Markandeswar College of Dental Sciences and Research, Ambala, Haryana, India; 4 Private Practitioner, Gurukripa Dental Care, Sector 28, Department of Prosthodontics, Chandigarh, India; 5 Department of Periodontics, Swargiya Dadasaheb Kalmegh Smruti Dental College and Hospital, Wanadongri, Hingna, Nagpur, Maharashtra, India; 6 Department of Paediatric and preventive dentistry, Rungta College of Dental Sciences and Research, Kohka, Bhilai, Chhattisgarh, India; 7 Intern, Kalinga Institute of Dental Sciences, Kalinga institute of Industrial Technology (KIT), Deemed to be university, Bhubaneswar, Odisha, India; 8 Department of Oral and Maxillofacial Surgery, Uttar Pradesh University of Medical Sciences, Saifai, Etawah, India

**Keywords:** Diode laser, defects, intrabony, probing pocket depth, recession

## Abstract

Patients with periodontitis frequently have intra-bony defects. Therefore, it is of interest to evaluate the use of a diode laser and a bone graft (nano hydroxyapatite [HA] + platelet rich fibrin [PRF]) in healing of intra-bony defects. In this split-mouth evaluation, 20 patients with bilateral intra-bony defects were divided into 2 groups as; Group I (control) - nano HA + PRF and Group II (Test group)--bone graft (nano HA + PRF)+ diode laser and treated accordingly. After six months, reductions in PI, PD, GI, GR, and RCAL were discovered. Intra-bony pockets were found to have significantly decreased in both groups.

## Background:

Intrabony defects are quite frequent and can be one, two, or three-wall defects depending on how many bony walls are still present [1, 2-3]. Periodontal therapy's primary objective is to regenerate lost periodontal tissue with improvement in health, and function of the gingiva, the alveolar bone, the cementum, and the periodontal ligament. Its role is to control the periodontal disease process by reducing infection and inflammation [1]. While treating intrabony defects with graft materials, it is necessary to remove bacteria from the intrabony pockets because they reduce success rates by impeding the regeneration process and the healing of osseous defects. Mechanical therapy alone, however, is insufficient to eradicate these bacteria [4]. Numerous materials, including allografts, xenografts, autogenous grafts, and alloplasts, have shown promise in the management of periodontal osseous defects both singly and in combination [2]. Porous hydroxyl+apatite (HA) bone graft material has been used to fill periodontal intrabony defects because of its exceptional bone conductive properties, which encourage the growth of osteogenic cells. Periodontal intrabony defects may be treated with a type of periodontal regenerative therapy that combines the use of porous HA, tricalcium phosphate (TCP), platelet rich fibrin (PRF) and platelet-rich plasma (PRP) [2, 3]. It was found that, PRP/BG combination and BG alone are efficient in the treatment of intra-bony defects [2]. Periodontitis is frequently treated with laser technology. In the management of peri-implantitis and periodontitis, it has been used to get rid of bacteria and pocket lining as well as get rid of endotoxins and calculus to improve periodontal tissue healing [4]. Diode lasers are a more effective way to manage osseous defects than scaling and root planing, according to a number of studies. Many studies have shown that, low laser therapy helps in healing infra bony defects [5, 6, 7-8]. Light Amplification by Stimulated Emission of Radiation is referred to as LASER. Based on the ideas of quantum physics, a laser is a device that produces a beam of light with all of the photons in a coherent state, typically with the same frequency and phase. Both how the laser energy is supplied to the surgical site and how it affects the tissue depend on its wavelength. The wavelength of the laser is between 300 and 2400 nm. A stimulated active medium is the source of a laser's monochromatic, unidirectional, coherent emission. Laser energy can be absorbed, scattered, or reflected when it strikes a target tissue. In dental surgery, this phenomenon is employed to slice or sterilize tissue. For dental applications, lasers come in diode, Nd:YAG, and Nd:YAP varieties [9]. Therefore, it is of interest to assess the efficacy of diode laser with PRF graft on intrabony defect healing after treatment.

## Materials and Methods:

## Sample size estimation:

A minimum of 18 sites per quadrant were required to achieve 80% power, a two-sided alpha level of 0.05, and a pocket depth of 1 mm to effective size reduction. Hence 20 patients were enrolled in this trial in order to account for potential dropouts.

## Inclusion and exclusion criteria:

Systemically healthy patients with bilateral infra bony defect, good oral hygiene (defined as a whole-mouth plaque index [PI]), pockets deeper than 6 mm after the initial therapy, patents not under any medication and who gave consent for participation in age group of 30-55 years of both genders were included for the study. Pregnant women, patient with any systemic and genetic conditions were excluded from the study. The current research was conducted in the Periodontics department after receiving approval from the Institutional Ethics Committee and obtaining the patients' written informed permission. This split-mouth study with bilateral intrabony defects was done by a single trained investigator from April 2018 to February 2020. In each patient the test and control quadrant group was assigned using computer-generated randomization sheet. All subjects' demographic information (name, gender, age, etc.) was recorded prior to study.Prior to procedure, five millilitres of blood was collected from each participant. These samples were centrifuged at 30.000 RPM for 15 minutes to create autologous PRF. One piece of PRF was cut into pieces and combined with the Nano-HA reinforced Fisiograft, while the other piece was picked up and compressed between two sterile glass slides to form thin membrane. Local anaesthesia was established using Lignocaine 2% with 1:100,000 Epinephrine. Using facial and palatal/lingual sulcular incisions, the mucoperiosteal flaps that extended from a tooth, both mesial and distal, to the study tooth were elevated. Additionally, root planning and defect debridement were done using hand curettes and ultrasonic tools; osseous recontouring was not done. The defects on one side of the arch were treated with a laser application [diode laser (Picasso Lite Laser -AMD Lasers, Inc., dental laser technology, Alan Miller Company) with an 810 nm wavelength, 2.5 W maximum output power, and 30 ms pulse duration] followed by a bone graft (FISIOGRAFT NanoHA-reinforced bone graft (HA) + PRF), while defects on the other side were treated with the same bone graft alone without laser (control group). In laser group continuous wave lasting 20 seconds, the power was set at 0.8 W. A fibre placed on the tissue at the top of the periodontal pocket, which moved towards the bottom of the pocket, diverted the laser energy away from the tooth structure. As it was moved both vertically and horizontally, this fibre maintained contact with the soft tissue lining of the pocket. The flaps were sutured with a 3-0 black braided silk suture using the interrupted stitch technique to achieve maximum closure. A COE-Pack was marketed as a periodontal pack, in addition. All necessary patients were given analgesics, antibiotics, and 0.12% chlorhexidine gluconate mouthwash three times a day for five days. A single calibrated assessor used a University of North Carolina-15 probe to perform all periodontal measurements. At baseline, three months later, and six months later, all patients' clinical and radiologic measurements such as; gingival index (GI) (Silness and Loe, 1963) and the PI (Silness and Loe, 1967) were noted. For probing depth (PD), gingival recession (GR), and relative clinical attachment level (RCAL), site-specific measurements were made. Additionally, the apical end of the stent, the gingival margin, and the GR were used as references [4]. Furthermore, a millimetre grid was used to conduct a parallel radiographic assessment. For each case, the radiographic parameter was set to 70 kVp, 8 mA, and an exposure time of 0.8 s. The difference between the pre- and post-operative radiographic distance between the cement enamel junction and the base of the intrabony defect was computed to track the hard-tissue fill. In order to collect data, more boxes with hard-tissue fill were added to the grid of boxes, each measuring 1 mm in height and width. The IBM Statistical Package for the Social Sciences statistical software, version 23 (Chicago, USA), was used to tabulate the results and perform statistical analysis using the Mann-Whitney U test. It was deemed significant if P 0.05.

## Results:

The mean SD for PI (mm) was 0.73 0.14 at baseline, 0.71 0.12 after three months, and 0.58 0.15 after six months, according to Table 1. At baseline, the mean SD for GI (mm) was 1.75 0.54; at three months, it was 1.14 0.19; and at six months, it was 0.62 0.18. Between baseline and six months after treatment, there was a significant drop in PI and GI for the intra-group. From baseline to the three- and six-month post-treatment points, the probing pocket depth (PPD) decreased. The difference in both groups was statistically considerable (0.01). The inter-group comparison was significance (Table 2). The control group's mean SD PPD (mm) values were 5.23 1.13 at baseline, 3.22 0.58 at three months, and 2.74 0.61 at six months. Additionally, the test group II's mean and standard deviation for PPD (mm) were 5.621.30 at baseline, 3.03 0.32 at three months, and 2.52 0.38 at six months. Mean PI and GI were decreased from baseline to 6 months (Table 1). Evaluation of mean PPD was statistically significant for intra group II and inter group at baseline and after 3 month duration (p<0.01) (Table 2). In group I, the mean SD for GR was 3.61 1.32 at baseline, 3.18 1.17 after three months, and 3.22 1.41 after six months, according to Table 3. In the test group II, the mean and SD for GR was 3.520.75 at baseline, 3.111.31 at three months, and 3.081.21 at six months. The comparison between the groups was significance. In group I, the mean SD for RCAL was 9.53 1.64 at baseline, 8.32 1.13 after three months, and 7.54 1.34 after six months, according to Table 4. RCAL in the test group had a mean and standard deviation of 9.85 1.58 at baseline, 7.34 1.87 at three months, and 6.35 1.51 at six months. Both inter and intra group comparison for PI, GI, probing pocket depth gingival recession and clinical attachment level was significant and it was better in test group with laser compared to group I. Gingival recession was statistically significant for intra group comparison in group II and after 6 month for inter group comparison (P<0.01) (Table 3). CAL was statistically significant for inter group comparison (Table 4). A millimetre grid measurement at baseline and after 6 months of follow up showed improvement in bone height in both the group and comparatively greater bone fill with laser group compared to graft alone.

## Discussion:

Osseous defects can be intra-bony, sub-bony, supra-bony or supra-crestal. If a pocket's base is coronal or occlusal to the bone crest, it is referred to as 'supra-bony'. However, if the pocket's apical end is below the bone crest, it is described as 'intra-bony' [10]. Intrabony defects come in two varieties: crater and defect. A sub-crestal component that affects the root surfaces of two adjacent teeth equally is referred to as a "crater verity," whereas a component that only affects the root sulci of one tooth is referred to as a "defect verity" [11]. Periodontal pathogens must be completely eradicated from intra-bony pockets for full healing to take place [12]. The goal of the current research was to evaluate how well intra-bony defects healed after bone grafting and laser treatment. According to Attia *et al.* treating intra-bony defects with low-level diode laser therapy is advantageous [13]. When Kamma *et al.* evaluated the efficacy of diode lasers for patients with aggressive periodontitis, they discovered that after six months, the Treponema denticola and Porphyromonas gingivalis levels were significantly lower in the scaling and root planing (SRP) and diode laser treatment (LAS) group. Additionally, the clinical attachment level slightly increased while PD decreased in the SRP + LAS group [14]. Diode lasers are also effective at lowering PPD and raising bone in patients with peri-implantitis, according to Roncati *et al.* [15]. In contrast to our findings, Gangolu *et al.* found that, additional laser irradiation at the test site did not exhibit any significant benefits in the bone regeneration [16]. Additionally, Gupta *et al.* came to the conclusion that the technique did not improve the healing of intra-bony defects treated with BG after evaluating the use of a diode laser for pocket sanitization prior to the implantation of bone biomaterial [4]. Additionally, the study by Naqvi *et al.* revealed that BG putty works well for treating intra-bony defects both on its own and in conjunction with PRF [17]. The majority of researches on periodontal defects have focused on HA. Synthetic HA is biocompatible, osteoconductive, and osteophilic, and shares a close but not exact structural and chemical resemblance with bone mineral. BG is capable of forming a chemical bond with living hard tissues by forming a carbonated HA surface layer on their surface. A silica-rich gel covers BG when it is exposed to tissue fluid. Additionally, a layer rich in calcium phosphate is formed, which promotes osteoblast cell absorption and concentration, leading to the formation of an extracellular matrix and mineralization [18]. Kim *et al.* claim that BG encourages and controls osteogenesis and permits rapid bone formation [19]. Ong *et al.* did not discover any proof indicating the importance of BG in the treatment of periodontal defects, though [20]. Behdin *et al.* conducted a systematic review and meta-analysis. They discovered lasers are less effective than conventional approaches at promoting healing and regeneration [21]. In addition, Soares *et al.* discovered that, while high-level laser therapy does not appear to enhance the effect of enamel matrix derivative in the regeneration process, low-level laser therapy has a positive impact on the regeneration of periodontal tissues [22]. In comparison to LLLT alone or PRF+NanoHA alone, Hemaid *et al.* reported that the combination of LLLT+PRF+NanoHA as a treatment modality produced the best results in bone formation in the bone defect. This result is similar to our findings [23]. Mathur *et al.* found that, PRF or ABG were effective in the treatment of infrabony defects [24]. According to Bhatia *et al.* the use of PRP in conjunction with a bone graft to treat intrabony defects is safe and results in better defect fill [25]. In the current study, it was discovered that the bone graft and the diode laser and graft combination were both successful in treating infrabony defects. We found significant effect with laser and bone graft combination compared to control group. It was mainly due to effect of laser in irradicating microorganism. The application of lasers and the reduction of bony defects made possible by the bone graft aid in the removal of microorganisms from pockets. All of the patients kept up with their oral hygiene throughout the entire investigation and none of them experienced any negative side effects from the graft material either during or after the procedure. This study's use of a small sample size was one flaw. As a result, it was impossible to assess the effects of laser pocket debridement with certainty. To confirm the findings and assess the true effects of laser therapy on periodontal tissues that have undergone regeneration, additional studies using larger samples are necessary.

## Conclusion:

Both the bone graft (HA + -PRF) with diode lasers combination was more effective in treating infrabony defects compared to use of HA+PRF alone due to additional effect of laser in improving the bony pocket. Additionally, it was found that the PRF was helpful in reducing bony defects.

## Figures and Tables

**Table 1:** Evaluation of mean plaque index and gingival index

**Time interval**	**PI (Mean ± SD)**	**GI (Mean ± SD)**
Baseline	0.73± 0.14	1.75±0.54
After three months	0.71± 0.12	1.14± 0.19
After six months	0.58± 0.15	0.62± 0.18
Change of PI from baseline to after three months	0.02± 0.02	1.61± 0.35
Change of PI from baseline to after six months	0.15±0.01	1.13± 0.36
SD = standard deviation

**Table 2:** Evaluation of mean probing pocket depth (in mm)

	**Time interval**					**P-value**
Groups	Baseline	After three months	After six months	Change of PPD from baseline to after three months	Change of PPD from baseline to after six months	
Group I (bone graft alone)	5.23± 1.13	3.22± 0.58	2.74± 0.61	2.01± 0.55	2.49±1.13	0.03
Group II (bone graft +laser)	5.62±1.30	3.03± 0.32	2.52± 0.38	2.59± 0.98	3.10± 0.92	0.01
Difference	0.39±0.17	0.19±0.26	0.22±0.23	-0.58± 0.43	-0.61± 0.21	
P-value	0.01	0.01	0.02	0.03	0.03	
P <0.05,
SD = standard deviation

**Table 3:** Evaluation of mean gingival recession (in mm)

**Time interval**	**Group I**	**Group II**	**Difference**	**P-value**
Baseline	3.61± 1.32	3.52±0.75	0.09±0.57	0.04
After three months	3.18± 1.17	3.11± 1.31	0.07± 0.14	0.03
After six months	3.22± 1.41	3.08± 1.21	0. 14±0.20	0.01
Change of GR from baseline to after three months	0.43± 0.15	0.41± 0.56	0.02± 0.41	0.04
Change of GR from baseline to after six months	0.39±0.09	1.44± 0.46	1.05± 0.37	0.01
P-value	0.02	0.01		
P <0.05,
SD = standard deviation

**Table 4:** Evaluation of mean relative clinical attachment level (in mm)

	**Time interval**					**P-value**
Groups	Baseline	After three months	After six months	Change of RCAL from baseline to after three months	Change of RCAL from baseline to after six months	
Group I (bone graft)	9.53± 1.64	8.32± 1.13	7.54± 1.34	1.21± 0.51	1.99± 0.30	0.03
Group II (bone graft +laser)	9.85±1.58	7.34± 1.87	6.35± 1.51	2.51± 0.29	3.50± 0.07	0.01
Difference	-0.32±0.06	0.98± 0.74	1.19± 0.17	-1.30± 0.22	-1.51± 0.23	
P-value	0.01	0.01	0.01	0.02	0.02	
p <0.05,
SD = standard deviation

## References

[1] Gerhardt L.C, Boccaccini A.R (2010). Materials (Basel)..

[2] Demir B (2007). J Clin Periodontol..

[3] Kaushick B.T (2011). Indian J Dent Res..

[4] Gupta R (2019). J Indian Soc Periodontol..

[5] Moritz A (1998). Lasers Surg Med..

[6] Kreisler M (2005). Lasers Surg Med..

[7] Aoki A (2015). Periodontol 2000..

[8] Mombelli A (2000). J Periodontol..

[9] Yeragi E (2019). (IOSR-JDMS)..

[10] Raffetto N (2004). Dent Clin North Am..

[11] Low S.B (1997). Int J Periodontics Restorative Dent..

[12] Froum S.J (1998). J Periodontol..

[13] Attia M.S (2019). InterAcad Periodontol..

[14] Kamma J.J (2009). Photomed Laser Surg..

[15] Roncati M (2013). J Indian Soc Periodontol..

[16] Gangolu M (2022). Journal of Clinical and Diagnostic Research..

[17] Naqvi A (2017). J Clin Diagn Res..

[18] Mistry S (2012). J Indian Soc Periodontol..

[19] Kim C.K (1998). J Periodontol..

[20] Ong M.A (1998). J Periodontol..

[21] Behdin S (2015). J Periodontol..

[22] Soares D.M (2014). Revista Clínica de Periodoncia, Implantología y Rehabilitación Oral..

[23] Hemaid S (2019). Open Access Maced J Med Sci..

[24] Mathur A (2015). Eur J Dent..

[25] Bhatia G (2018). Indian J Dent Sci..

